# USP5 promotes lipopolysaccharide-induced apoptosis and inflammatory response by stabilizing the TXNIP protein

**DOI:** 10.1097/HC9.0000000000000193

**Published:** 2023-08-03

**Authors:** Songchang Shi, Xiaobin Pan, Minyong Chen, Lihui Zhang, Shujuan Zhang, Xincai Wang, Songjing Shi, Zhixin Chen, Wei Lin, Yi Jiang

**Affiliations:** 1 Department of Critical Care Medicine, Shengli Clinical Medical College of Fujian Medical University, Fujian Provincial Hospital South Branch, Fujian Provincial Hospital, Fuzhou, Fujian Province, China; 2 Department of Hepatobiliary and Pancreatic Surgery, Clinical Oncology School of Fujian Medical University, Fujian Cancer Hospital, Fuzhou, Fujian Province, China; 3 Department of Critical Care Medicine, Shengli Clinical Medical College of Fujian Medical University, Fujian Provincial Hospital, Fuzhou, Fujian Province, China; 4 Fujian College Association Instrumental Analysis Center of Fuzhou University, Fuzhou, Fujian Province, China; 5 Department of Endocrinology, Shengli Clinical Medical College of Fujian Medical University, Fujian Provincial Hospital, Fuzhou, Fujian Province, China; 6 Department of Hepatobiliary and Pancreatic Surgery, Fuzong Clinical Medical College of Fujian Medical University, 900 Hospital of the Joint Logistics Team, PLA, Fuzhou, Fujian Province, China

## Abstract

**Background:**

The role of thioredoxin-interacting protein (TXNIP) in lipopolysaccharide-induced liver injury in mice has been reported, but the underlying mechanisms are poorly understood.

**Methods:**

We overexpressed deubiquitinase in cells overexpressing TXNIP and then detected the level of TXNIP to screen out the deubiquitinase regulating TXNIP; the interaction between TXNIP and deubiquitinase was verified by coimmunoprecipitation. After knockdown of a deubiquitinase and overexpression of TXNIP in Huh7 and HepG2 cells, lipopolysaccharide was used to establish a cellular inflammatory model to explore the role of deubiquitinase and TXNIP in hepatocyte inflammation.

**Results:**

In this study, we discovered that ubiquitin-specific protease 5 (USP5) interacts with TXNIP and stabilizes it through deubiquitylation in Huh-7 and HepG2 cells after treatment with lipopolysaccharide. In lipopolysaccharide-treated Huh-7 and HepG2 cells, USP5 knockdown increased cell viability, reduced apoptosis, and decreased the expression of inflammatory factors, including NLRP3, IL-1β, IL-18, ASC, and procaspase-1. Overexpression of TXNIP reversed the phenotype induced by knockdown USP5.

**Conclusions:**

In summary, USP5 promotes lipopolysaccharide-induced apoptosis and inflammatory response by stabilizing the TXNIP protein.

## Graphical abstract

**Figure:**
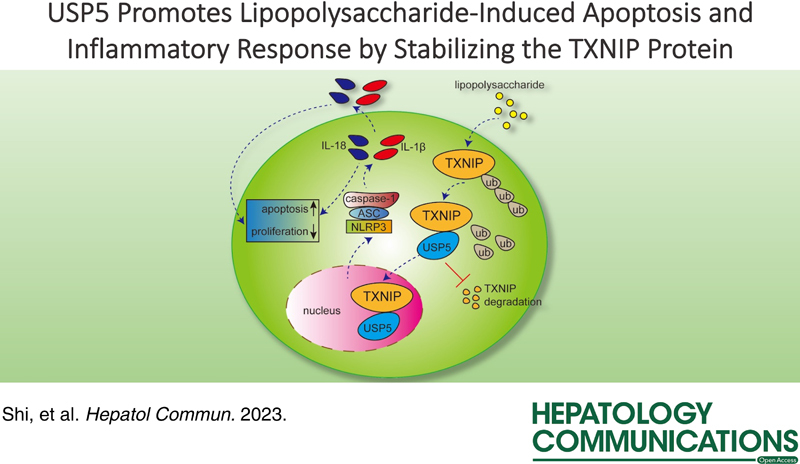

## INTRODUCTION

Sepsis-induced liver injury, which is regulated by several proteins, is a manifestation of multiple organ dysfunction. Thioredoxin-interacting protein (TXNIP), the most important regulatory thioredoxin (TRX)-binding protein in the body, induces oxidative stress, suppresses cell proliferation, and mediates cell apoptosis by inhibiting the function of the thioredoxin system.^1–3^ TXNIP is a core molecule in the inflammatory process due to insulin production, which leads to the death of insulin-producing cells in the human pancreas.^4^ TXNIP is a complex composed of TRX, thioredoxin reductase (TRX-R), and reduced coenzyme II (NADPH), which has redox activity.^5^ It mediates oxidative stress response by inhibiting TRX expression. Oxidative stress is an important pathway that activates NLRP3 and procaspase-1 inflammasome.^6,7^ It has been reported that inhibition of TXNIP-NLRP3 inflammasome could alleviate liver injury in mice after treatment with lipopolysaccharide (LPS).^8^

Protein activity is controlled through post-translational modifications, typically achieved through E1-E2-E3 enzymes.^9^ E3 ubiquitin ligases comprise 5 classes; they select substrates for ubiquitin conjugation, which is reversed by the action of deubiquitinating enzymes.^10^ One of these classes, Ub-specific protease (USP), has been shown to regulate the deubiquitinating pathway. USP11 modulates the polysome regulation activity of the INK4a tumor suppressor.^11^ USP7 is a novel regulator of Chk1 protein stability, which regulates the DNA damage response pathway.^12^ The deubiquitinating enzyme USP2 regulates the p53 pathway by targeting Mdm2.^13^ USP5 has been reported to target several proteins, such as SLUG, PD-L1, cyclin D1, and Cav3.2, promoting liver cancer progression, proliferation of glioblastoma cells, immune response, and inflammatory pain.^14–17^ However, the role of USP5 in sepsis-induced liver injury remains unclear.

Based on the previous description,^18,19^ LPs-induced Huh7 and HepG2 cells were used to establish cell models of septic liver injury to analyze the role of TXNIP-stabilizing USPs in the progression of sepsis-induced liver injury and in the NLRP3 inflammatory pathway.

## Methods

### Cell lines

Huh7 and HepG2 cells were obtained from Xiamen Immocell Biotechnology Co. Ltd. and individually maintained in DMEM (Procell, China) supplemented with 10% fetal bovine serum and 1% penicillin-streptomycin solution. Cells were cultured in an incubator at 37 °C with an atmosphere of 5% CO_2_.

### Plasmids

Plasmids encoding USP5, USP49, USP39, USP11, USP26, USP36, USP6, USP15, USP20, USP46, USP52, USP35, USP33, USPL1, OTUB1, USP2, USP30, USP7, USP40, USP22R, USP29, USP48, USP53, USP14, USP25, USP3, USP10, USP21, USP22, USP45, USP4, USP44, USP18, USP19, USP32, USP51, USP54, USP38, USP13, or USP8, pCDH-HA vector, pCDH-Flag vector, pLKO.1-puro vector, and plasmid His-ubiquitin (His-Ub) were obtained from XIAMEN Anti-hela Biological Technology Trade Co. Ltd., (Xiamen, China). The first cDNA was synthesized by cDNA synthesis kit (catalog number: R111-01, Vazyme, Nanjing, China) using RNA as the template. Specific primers and PCR kits (catalog number: P503-d1, Vazyme, Nanjing, China) were used to amplify cDNA. The cDNAs of USP5 and TXNIP were inserted into pCDH-HA vector and pCDH-Flag vector, respectively, and the resulting plasmids were named HA-USP5 and Flag-TXNIP, respectively. The short hairpin RNA (shRNA) of USP5 was inserted into the pLKO.1-puro vector to obtain a plasmid that encodes USP5 shRNA and was named shUSP5. The following primers were used: HA-USP5 forward primer: 5′-TAGAGAATTCGGATCCATGGCGGAGCTGAGTGAGGAGG-3′ and HA-USP5 reverse primer: 5′-GCTTCCATGGCTCGAGTTAGCTGGCCACTCTCTGGTAG-3′; Flag-TXNIP forward primer: 5′-GAAATGTACAAGGAATTATGGTGATGTTCAAGAAGATC-3′, Flag-TXNIP reverse primer-1: 5′-TCGTCATCGTCTTTGTAGTCCTGCACATTGTTGTTGAGG-3′, and Flag-TXNIP reverse primer-2: 5′-AATTTCTAGGGATCCTCACTTGTCGTCATCGTCTTTGTAGTC-3′; shUSP5-1 forward primer: 5′-CCGGCCTGTCTGTAAGGAGACTTTGCTCGAGCAAAGTCTCCTTACAGACAGGTTTTT-3′ and shUSP5-1 reverse primer: 5′-AATTAAAAACCTGTCTGTAAGGAGACTTTGCTCGAGCAAAGTCTCCTTACAGACAGG-3′; shUSP5-2 forward primer: 5′- CCGGGACCACACGATTTGCCTCATTCTCGAGAATGAGGCAAATCGTGTGGTCTTTTT-3′ and shUSP5-2 reverse primer: 5′- AATTAAAAAGACCACACGATTTGCCTCATTCTCGAGAATGAGGCA;AATC;GTGTGGTC-3′; and shUSP5-3 forward primer: 5′- CCGGGATAGACATGAACCAGCGGATCTCGAGATCCGCTGGTTCATGTCTATCTTTTT-3′ and shUSP5-3 reverse primer: 5′ AATTAAAAAGATAGACATGAACCAGCGGATCTCGAGATCCGCTGGTTCATGTCTATC-3′.

### Cell viability measurement

Huh7 or HepG2 cells were seeded into 96-well plates at a density of 8000 cells per well for 12 hours. Cells were incubated with 250, 500, 1000, 2000, or 4000 ng/mL LPS for 24 hours. Alternatively, cells were divided into 6 groups: MOCK (phosphate buffer solution), LPS, LPS+shNC, LPS+shUSP5, LPS+shUSP5+Vector, and LPS+shUSP5+TXNIP. After transfection with 0.2-µg plasmid (shNC, shUSP5, Vector, or TXNIP) for 24 hours, cells were incubated with 1000 ng/mL LPS for 24 hours. Subsequently, 5 mg/mL MTT (10 μL per well) was added and then incubated for an additional 4 hours. Subsequently, the supernatant was discarded and 150 μL DMSO was added. The absorbance of each well was then measured at 490 nm. Cell viability data were calculated and compared with that of untreated cells.

### Apoptosis detection using annexin V-FITC staining and flow cytometry

Huh7 or HepG2 cells were seeded into 6-well plates at a density of 100,000 cells per well for 12 hours. Cells were incubated with 1000 ng/mL LPS for 24 hours. Alternatively, cells were divided into 6 groups: MOCK (phosphate buffer solution), LPS, LPS+shNC, LPS+shUSP5, LPS+shUSP5+Vector, and LPS+shUSP5+TXNIP. After transfection with 5-µg plasmid (shNC, shUSP5, vector, or TXNIP) for 24 hours, 70% confluence of cells in 6-well plates were incubated with 1000 ng/mL LPS for 24 hours. Subsequently, the cells were collected through centrifugation at 1000 rpm for 5 minutes followed by 3 washes with PBS. Cell apoptosis was measured according to the manufacturer’s instructions (Annexin V-FITC kit, Beyotime Biotechnology, China). Annexin V-FITC staining solution, PI staining solution, and binding buffer were then added to the cells while mixing slowly. After incubation in the dark for 20 minutes at room temperature, apoptosis was immediately measured through flow cytometry.

### Enzyme-linked immunosorbent assay

Huh7 or HepG2 cells were seeded into 6-well plates at a density of 100,000 cells per well for 12 hours and were divided into 6 groups: MOCK (phosphate buffer solution), LPS, LPS+shNC, LPS+shUSP5, LPS+shUSP5+Vector, and LPS+shUSP5+TXNIP. After transfection with 5-µg plasmid (shNC, shUSP5, Vector, or TXNIP) for 24 hours, 70% confluence of cells in 6-well plates were incubated with 1000 ng/mL LPS for 24 hours. Subsequently, the supernatant was collected for IL-1β and IL-18 detection using IL-1β ELISA kit (CAT#: RLB00, R&D Systems, Hinnerup, Denmark) and IL-18 ELISA kit (CAT#: DY3144-05, R&D Systems) as described.^20^ The sample concentrations were calculated according to the standard curve obtained.

### Real-time PCR analysis

Total RNA from cells was extracted using TRIzol reagent (Invitrogen) and reverse-transcribed into cDNA using a reverse transcription kit (Catalog number: RR036A, TAKARA). Quantitative PCR was performed using the StepOnePlus Real-Time PCR system (Applied Biosystems, USA) and TB Green Premix Ex Taq ™II (TaKaRa, Dalian, China) with specific primers for the detected genes. The RNA content was analyzed using the comparative threshold cycle method. The plasmid construction and primer sequences are listed in as follow: 18s RNA forward primer: 5′-AGGCGCGCAAATTACCCAATCC-3′ and 18s RNA reverse primer: 5′-GCCCTCCAATTGTTCCTCGTTAAG-3′; USP5 forward primer: 5′-GAAGTGTTCCGCTTCTTGGTGG-3′ and USP5 reverse primer: 5′-TTGCCGCTTCTTCTCCTCGTAC-3′; TXNIP forward primer: 5′-CAGCAGTGCAAACAGACTTCGG-3′ and TXNIP reverse primer: 5′-CTGAGGAAGCTCAAAGCCGAAC-3′; NLRP3 forward primer: 5′-GGACTGAAGCACCTGTTGTGCA-3′ and NLRP3 reverse primer: 5′-TCCTGAGTCTCCCAAGGCATTC-3′; ASC forward primer: 5′-GATGGAAGCATCCATTCAGCCTA-3′ and ASC reverse primer: 5′-CCACCAAGGTTCTCCTACTGTC-3′; caspase-1forward primer: 5′-GCTGAGGTTGACATCACAGGCA-3′, and caspase-1 reverse primer: 5′-TGCTGTCAGAGGTCTTGTGCTC-3′; IL-1βforward primer: 5′-CCACAGACCTTCCAGGAGAATG-3′ and IL-1β reverse primer: 5′-GTGCAGTTCAGTGATCGTACAGG-3′; and IL-18 forward primer: 5′-GATAGCCAGCCTAGAGGTATGG-3′ and IL-18 reverse primer: 5′-CCTTGATGTTATCAGGAGGATTCA-3′.

### Western blotting

The cells were collected and lysed in RIPA buffer with a phosphatase inhibitor on ice for 30 minutes. Samples were sonicated using Bioruptor for 10 minutes (30 s on and 30 s off). Proteins were extracted, and the protein concentration was quantified using a BCA kit (Cat. No. PA115-02; TIANGEN Biotechnology, Beijing, China). The samples were then boiled at 95°C for 10 minutes, and 20-μg protein was loaded per well of 10% SDS-PAGE gels. Electrophoresis was performed at 80 V for 1.5 hours, while electrophoretic transfer was performed at 300 mA for 2.5 hours. The PVDF membranes were blocked with 5% skim milk dissolved in TBS containing 0.05% Tween 20 (TBST) for 1 hour and incubated with TBST buffer containing β-actin antibody (catalog number: 20536-1-AP, Proteintech, Wuhan, China), TXNIP antibody (catalog number: 18243-1-AP, Proteintech), USP5 antibody (catalog number: 10473-1-AP, Proteintech), ASC antibody (catalog number: 10500-1-AP, Proteintech), NLRP3 antibody (catalog number: 27458-1-AP, Proteintech), Flag-Tag antibody (catalog number: 20543-1-AP, Proteintech), HA-tag antibody (catalog number: 51064-2-AP, Proteintech), His-tag antibody (catalog number: 10001-0-AP, Proteintech), GAPDH antibody (catalog number: 10494-1-AP, Proteintech), and β-actin overnight (catalog number: 20536-1-AP, Proteintech) at 4°C. The membranes were then incubated with 1:10000 dilutions of HRP-conjugated goat anti-mouse IgG (H+L) (Cat. No: SA00001-1, Proteintech) or goat anti-rabbit IgG (H+L) (Cat. No: SA00001-2, Proteintech) for 1 hour at 25°C. After 3 washes with TBST (10 min each wash), an ECL reagent was added to the membrane and the protein bands were visualized using a Bio-Rad ChemicDoc machine.

### Coimmunoprecipitation

Huh7 and HepG2 cells were cotransfected with Flag-TXNIP, HA-USP5, shUSP5, or His-Ub plasmids for 48 hours. Transfected cells or untransfected cells were then collected and lysed in an immunoprecipitation buffer and sonicated for 15 minutes. Afterward, the cell lysates were centrifuged at 12,000*g* for 15 minutes, and the supernatant was collected. Then, the cell lysates were incubated with anti-Flag (M2), anti-HA (M2) beads or specific antibodies at 4°C overnight on a roller. The precipitated beads were washed five times with immunoprecipitation buffer. Immunoprecipitants were resolved on SDS-PAGE protein loading buffer, and western blotting was performed to detect the interaction between TXNIP and USP5.

### Immunofluorescence assay

The cells were digested with trypsin and seeded on coverslips in a 24-well plate. When the cell density reached 60%, the plasmid HA-USP5 or/and Flag-TXNIP were transfected into cells. After 24 hours, the cells were fixed in a 4% formaldehyde solution for 5 minutes. The cells were then blocked with 10% bovine serum albumin (BSA) in PBS containing 0.05% Tween-20 (PBST) for 10 minutes. Antibodies (1:50 dilution in 10% BSA dissolved in PBST) against Flag and HA were added to cells and incubated overnight at 4 °C. The cells were then stained with a DAPI working solution after incubation with the corresponding secondary antibodies for 45 minutes and washed 3 times with PBST. Finally, the coverslips were dried and imaged using confocal fluorescence microscopy.

### Statistical analysis

Bar charts and statistical analyses were performed using GraphPad Prism software, version 8.0. Unpaired Student’s *t* test was used to compare differences between 2 groups of parametric data. One-way ANOVA was used to analyze the differences among multiple groups followed by Tukey’s post test. Western blotting results were analyzed through photography, and the intensity of the signals was quantified using ImageJ software.

## RESULTS

### Screening for USP family genes that regulate TXNIP protein stability

**FIGURE 1:**
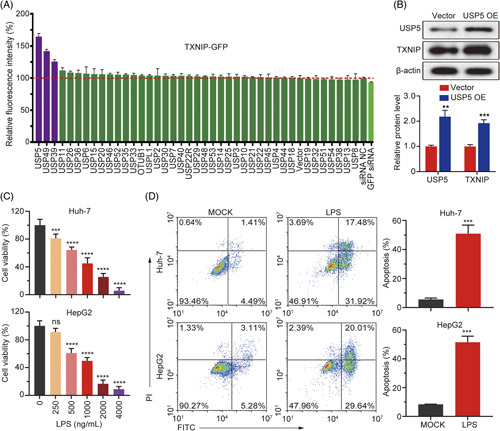
Screening for USP family genes that regulate TXNIP protein stability and establishment of a sepsis-induced liver injury model. (A) The fluorescence intensity in cells overexpressing TXNIP and USP protein. (B) TXNIP and USP5 expressions were detected through western blotting in USP5-overexpressing cells. (C) Establishment of a sepsis-induced liver injury cell model through LPS treatment. Huh7 and HepG2 cells were treated with 0, 250, 500, 1000, 2000, or 4000 ng/mL of LPS. Cell viability was detected using an MTT assay. (D) Cell apoptosis rate in Huh7 and HepG2 cells was monitored through flow cytometry using an Annexin V-FITC/PI Kit. **p < 0.01; ***p < 0.001; ****p < 0.0001. Abbreviations: LPS, lipopolysaccharide; ns, not significant; TXNIP, thioredoxin-interacting protein; USP, Ub-specific protease.

To understand which USP subunit could regulate TXNIP gene stability in cells, we overexpressed USP in 293T cells that overexpressed TXNIP-GFP protein, and found that the fluorescence was strongest in cells that overexpressed USP5 and TXNIP-GFP, indicating that USP5 may increase the level of TXNIP protein (Figure 1A). In addition, we investigated the correlation between the expressions of TXNIP and USP5 proteins by overexpressing USP5 in Huh7 and HepG2 cells. TXNIP protein was highly expressed in USP5-overexpressing cells compared with cells transfected with the empty vector (Figure 1B).

### Establishment and validation of a cell model of sepsis-induced liver injury

The viabilities of Huh7 and HepG2 cells were measured after they were treated with 0, 250, 1000, 2000, or 4000 ng/mL LPS (Figure 1C). Our results show that LPS significantly inhibited cell proliferation. Therefore, we used 1000 ng/mL LPS as a trigger for sepsis-induced liver injury in Huh7 and HepG2 cells. Similar to the cell viability data, half of the cells were apoptotic on treatment with 1000 ng/mL LPS (Figure 1D).

### USP5 regulates sepsis-induced liver injury

**FIGURE 2:**
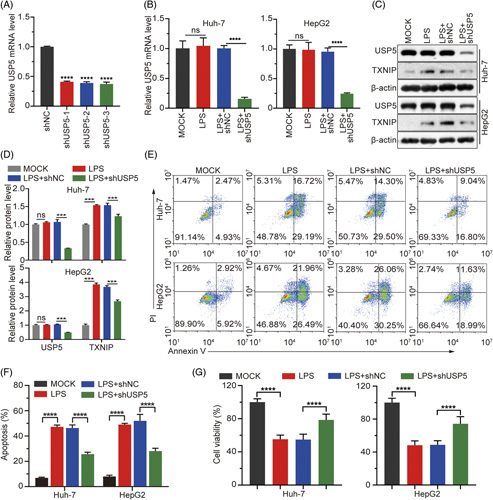
Effect of USP5 knockdown on a sepsis-induced liver injury cell model. (A and B) mRNA expressions of USP5 were detected through RT-qPCR in Huh7 and HepG2 cells. (C and D) Protein expressions of TXNIP and USP5 were measured through western blotting. (E) Cell apoptosis rate was monitored through flow cytometry using an Annexin V-FITC/PI Kit. (F) Quantitation of the apoptosis rate. (G) Cell viability was detected using an MTT assay. *** *p* < 0.001, **** *p* < 0.0001. Abbreviations: LPS, lipopolysaccharide; ns, not significant; TXNIP, thioredoxin-interacting protein; USP, Ub-specific protease.

To investigate the role of USP5 in sepsis-induced liver injury, USP5 knockdown was performed in LPS-treated Huh7 and HepG2 cells (Figures 2A, B). There were no significant differences in USP5 mRNA and protein levels in LPS-treated cells (Figure 2B–D). In contrast, the protein level of USP5 was effectively downregulated after treatment with USP5 shRNA (Figure 2B–D). Moreover, TXNIP was highly expressed in Huh7 and HepG2 cells after LPS treatment, which was inhibited by USP5 knockdown (Figure 2B–D). This further indicated that TXNIP might be regulated by USP5.

**FIGURE 3:**
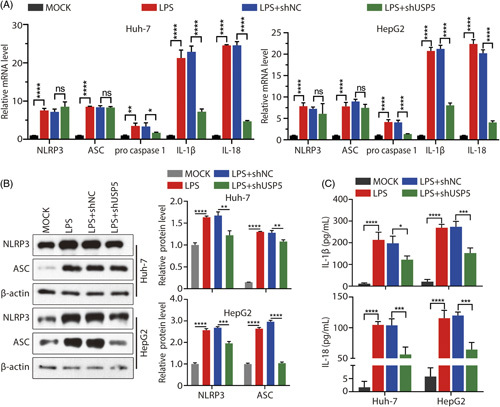
Detection of inflammatory cytokines after USP5 knockdown. (A) mRNA expressions of ASC, IL-18, NLRP3, IL-1β, and procaspase-1 were detected through RT-qPCR in Huh7 and HepG2 cells. (B) Protein expression of NLRP3 or ASC was detected through western blotting. (C)Quantitation of IL-1β and IL-18 levels in Huh7 and HepG2 cells as measured through ELISA. *p < 0.05; **p < 0.01; ***p < 0.001, ****p < 0.0001. Abbreviations: LPS, lipopolysaccharide; ns, not significant; TXNIP, thioredoxin-interacting protein; USP, Ub-specific protease.

To explore whether USP5 knockdown could alleviate sepsis-induced liver injury, cell viability and apoptosis rates were detected in LPS-treated cells transfected with a plasmid encoding USP5 shRNA. The results illustrated that USP5 knockdown could protect cells from LPS-induced injury by reducing the cell apoptosis rate and increasing cell viability (Figure 2E–G). Sepsis-induced liver injury is associated with inflammation. Therefore, we explored the levels of IL-18 and IL-1β through ELISA and measured the mRNA expression of proinflammatory factors, including ASC, IL-18, IL-1β, NLRP3, and procaspase-1 in Huh7 and HepG2 cells after LPS treatment or USP5 knockdown. The results revealed a robust increase in the expression of inflammatory factors in LPS-induced cell injury (Figure 3A–C). Interestingly, the upregulation of caspase 1, IL-1β, and IL-18 mRNA levels was decreased by USP5 knockdown, while the upregulation of ASC and NLRP3 mRNA levels was not decreased by USP5 knockdown (Figure 3A). However, elevated NLRP3 and ASC protein levels were inhibited by USP5 knockdown (Figure 3B). The levels of IL-1β and IL-18 were also inhibited by USP5 knockdown (Figure 3C). These results suggest that USP5 knockdown may rescue LPS-induced liver cell injury by inhibiting the expression of ASC, IL-18, IL-1β, and NLRP3.

### USP5 interacts and stabilizes TXNIP protein in cells

**FIGURE 4:**
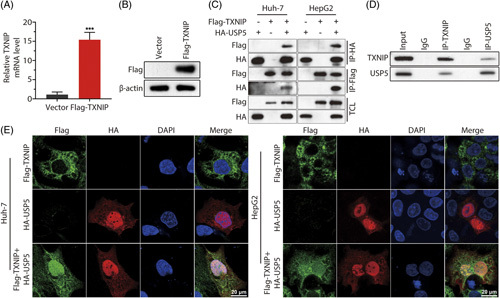
USP5 interacts with TXNIP. (A and B) mRNA and protein expression levels of TXNIP were detected after overexpressing TXNIP in cells. (C). Huh7 and HepG2 cells were cotransfected with HA-USP5 and Flag-TXNIP plasmids. Cell lysates were prepared for Co-IP with anti-Flag or anti-HA antibody. (D) Cell lysates were prepared for Co-IP with anti-TXNIP or anti-USP5 antibody. (E). Immunofluorescence staining of Huh7 and HepG2 cells after transfection with HA-USP5 and Flag-TXNIP plasmids. ***p< 0.001. Abbreviations: TXNIP, thioredoxin-interacting protein; USP, Ub-specific protease.

To explore the relationship between USP5 and TXNIP, we cotransfected Huh7 and HepG2 cells with plasmids encoding Flag-TXNIP and HA-USP5. The effective overexpression of TXNIP was detected at the mRNA and protein levels after transfection (Figure 4A, B). Moreover, a reciprocal co-IP assay showed that TXNIP was able to immunoprecipitate USP5 (Figure 4C). Moreover, the endogenous coimmunoprecipitation showed that TXNIP interacted with USP5 (Figure 4D). Finally, immunofluorescence analysis of Huh7 and HepG2 cells showed that USP5 and TXNIP colocalized in the nucleus (Figure 4E). In conclusion, these results indicate that USP5 forms a complex with TXNIP and exerts its function in the nucleus.

**FIGURE 5:**
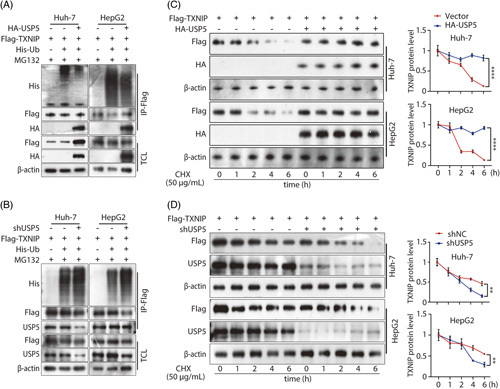
USP5 could stabilize TXNIP protein in cells. (A) The ubiquitinated forms of TXNIP were assayed through Co-IP and western blotting after transfection of HA-USP5, His-Ub, or Flag-TXNIP plasmids in Huh7 and HepG2 cells. (B) The ubiquitinated forms of TXNIP were assayed through Co-IP and western blotting after transfection of shRNA USP5, His-Ub, or Flag-TXNIP plasmids in Huh7 and HepG2 cells. (C) The half-life of TXNIP protein was detected in TXNIP-overexpressing cells transfected with vector or HA-USP5 plasmid after being treated with 50 μg/mL CHX for different durations. (D) The half-life of TXNIP protein was detected in TXNIP-overexpressing cells transfected with shNC or shUSP5 plasmid after being treated with 50 μg/mL CHX for different durations. Abbreviations: TXNIP, thioredoxin-interacting protein; USP, Ub-specific protease.

To explore whether USP5 mediates TXNIP ubiquitination, we performed *in vitro* ubiquitination assays in Huh7 and HepG2 cells. USP5 overexpression reduced TXNIP ubiquitination, while USP5 knockdown accelerated TXNIP ubiquitination in cells (Figure 5A, B). To investigate whether the expression of USP5 augments TXNIP by prolonging its half-life, the protein content of TXNIP was measured in the presence of the protein synthesis inhibitor cycloheximide (CHX). The half-life of TXNIP was longer in USP5-overexpressing cells than in the control cells (Figure 5C). Consistent with this, the half-life of TXNIP was shorter in USP5-knockdown cells than in control cells (Figure 5D). Taken together, these results indicate that USP5 stabilizes TXNIP by suppressing its ubiquitination.

### USP5 affects LPS-induced liver cell damage by regulating the stability of TXNIP protein

**FIGURE 6:**
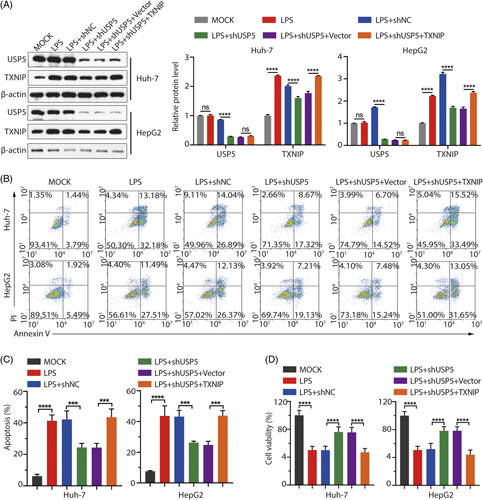
Knockdown of USP5 aggravates LPS-induced liver cell injury by regulating the stability of TXNIP protein. (A). Protein expression levels of TXNIP and USP5 were detected through western blotting. (B) Cell apoptosis rate was detected through flow cytometry using an Annexin V-FITC/PI Kit. (C) Quantitation of the apoptosis rate. (D) Cell viability was detected using an MTT assay. **p < 0.01; ***p < 0.001, ****p < 0.0001. Abbreviations: LPS, lipopolysaccharide; ns: not significant; TXNIP, thioredoxin-interacting protein; USP, Ub-specific protease.

**FIGURE 7:**
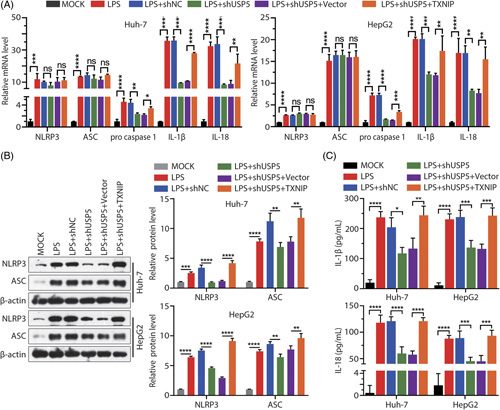
USP5 stabilizes TXNIP to promote the secretion of inflammatory factors. (A) mRNA expressions of ASC, IL-1β, IL-18, NLRP3, and procaspase-1 were detected through RT-qPCR in Huh7 cells after USP5 knockdown or TXNIP overexpression. (B) Protein expression levels of NLRP3 or ASC were detected through western blotting after USP5 knockdown or TXNIP overexpression. (C) Quantitation of IL-1β and IL-18 levels in Huh7 and HepG2 cells as measured using an ELISA Kit. *p < 0.05; **p < 0.01; ***p<0.001, ****p < 0.0001. Abbreviations: LPS, lipopolysaccharide; ns, not significant; TXNIP, thioredoxin-interacting protein; USP, Ub-specific protease.

To determine the functional significance of USP5-mediated TXNIP protein stabilization, we reduced USP5 protein level and overexpressed TXNIP protein level after cells treated with LPS (Figure 6A). Then, we examined the effect of reduced USP5 levels and overexpression of TXNIP on cell apoptosis. As shown in Figure 6B, C, concurrent USP5 depletion and TXNIP overexpression significantly increased apoptosis compared with USP5 depletion alone. Similarly, cell viability was lower than that in cells with USP5 depletion alone (Figure 6D). Finally, we examined the changes in mRNA expression of the inflammatory factors ASC, IL-18, IL-1β, procaspase-1, and NLRP3. The results showed that the mRNA levels of IL-18, IL-1β, and procaspase-1 decreased after knockdown of USP5 in LPS-induced sepsis cell model, and overexpressed TXNIP in USP5 knockdown cells could upregulate the inflammatory factors (Figure 7A). In LPS-induced sepsis cell models, there was no significant difference in mRNA levels of ASC and NLRP3, whether TXNIP was overexpressed with USP5 knockdown or USP5 was knocked down (Figure 7A). LPS-induced NLRP3, ASC, IL-18, and IL-1β protein levels were decreased by USP5 knockdown; overexpression of TXNIP suppressed the effect of USP5 knockdown in the cells (Figures 7B, C). These results further suggest that USP5 promotes LPS-induced apoptosis and inflammation by regulating TXNIP protein stability.

## DISCUSSION

Liver injury is associated with severe complications of sepsis, and sepsis-induced liver injury has been identified as a robust predictor of mortality.^21^ Although the mechanism of liver injury during sepsis remains unknown, the inflammatory response is known to play an important role in its progression. It has been reported that modulating TXNIP protein stability can suppress cell proliferation and mediate cell apoptosis by increasing the expression of inflammatory factors.^22,23^ The regulation of TXNIP/NLRP3 expression is considered to attenuate sepsis-induced lung injury and myocardial dysfunction.^24–26^ Therefore, modulating TXNIP protein stability might efficiently alleviate the inflammatory response to sepsis-induced liver injury and subsequently protect cells from injury.

LPS treatment has been used in inducing sepsis-induced liver injury for >40 years.^27^ Here, we found that LPS treatment inhibited cell viability and induced cell death at different doses. Next, we identified TXNIP deubiquitination, mediated by the deubiquitinating enzyme USP5, as a novel mediator of sepsis-induced liver injury. This was followed by the knockdown of USP5 for promoting cell proliferation, induction of cell apoptosis, and reduction in the mRNA and protein expression levels of inflammatory factors, including ASC, IL-1β, IL-18, NLRP3, and procaspase-1. Therefore, USP5 plays a key role in the downregulation of NLRP3 and procleaved caspase-1 activation by modulating TXNIP stability.

Although our study suggests that the knockdown USP5 could be an effective method for alleviating sepsis-induced liver injury by reducing TXNIP stability, several limitations should be considered. One limitation of this study is the establishment of a cell model. Huh7 and HepG2 cells are immortalized cell lines derived from human hepatoma cells and are widely used for liver injury research.^28^ Hence, it is necessary to establish animal models to ensure the accuracy of the results we obtained. Another limitation is that the LPS model, which was built on an elementary systemic inflammatory challenge, lacks an infectious point. Therefore, further validation of other sepsis models is necessary. For example, mice using puncture or *Klebsiella pneumoniae* have also been used as an *in vivo* sepsis model.^29^ An interesting point of our research that requires in-depth investigation is the physiological criteria that dynamically mediate TXNIP protein stability.

In summary, USP5 interacts with TXNIP and regulates its stability to promote LPS-induced liver cell injury by modulating the inflammatory response. Therefore, targeting USP5/TXNIP may be an important therapeutic strategy for sepsis-mediated liver injury.
